# Identification and Modulation of the Key Amino Acid Residue Responsible for the pH Sensitivity of Neoculin, a Taste-Modifying Protein

**DOI:** 10.1371/journal.pone.0019448

**Published:** 2011-04-29

**Authors:** Ken-ichiro Nakajima, Kanako Yokoyama, Taichi Koizumi, Ayako Koizumi, Tomiko Asakura, Tohru Terada, Katsuyoshi Masuda, Keisuke Ito, Akiko Shimizu-Ibuka, Takumi Misaka, Keiko Abe

**Affiliations:** 1 Department of Applied Biological Chemistry, The University of Tokyo, Tokyo, Japan; 2 Department of Nutritional Science, Tokyo University of Agriculture, Tokyo, Japan; 3 Agricultural Bioinformatics Research Unit, Graduate School of Agricultural and Life Sciences, The University of Tokyo, Tokyo, Japan; 4 Suntory Institute for Bioorganic Research, Osaka, Japan; 5 Food Safety and Reliability Project, Kanagawa Academy of Science and Technology, Kawasaki, Japan; University of Oldenburg, Germany

## Abstract

Neoculin occurring in the tropical fruit of *Curculigo latifolia* is currently the only protein that possesses both a sweet taste and a taste-modifying activity of converting sourness into sweetness. Structurally, this protein is a heterodimer consisting of a neoculin acidic subunit (NAS) and a neoculin basic subunit (NBS). Recently, we found that a neoculin variant in which all five histidine residues are replaced with alanine elicits intense sweetness at both neutral and acidic pH but has no taste-modifying activity. To identify the critical histidine residue(s) responsible for this activity, we produced a series of His-to-Ala neoculin variants and evaluated their sweetness levels using cell-based calcium imaging and a human sensory test. Our results suggest that NBS His11 functions as a primary pH sensor for neoculin to elicit taste modification. Neoculin variants with substitutions other than His-to-Ala were further analyzed to clarify the role of the NBS position 11 in the taste-modifying activity. We found that the aromatic character of the amino acid side chain is necessary to elicit the pH-dependent sweetness. Interestingly, since the His-to-Tyr variant is a novel taste-modifying protein with alternative pH sensitivity, the position 11 in NBS can be critical to modulate the pH-dependent activity of neoculin. These findings are important for understanding the pH-sensitive functional changes in proteinaceous ligands in general and the interaction of taste receptor–taste substance in particular.

## Introduction

Humans are able to sense sweetness when tasting a variety of compounds, including sugars, amino acids, peptides, glycosides and sweet-tasting proteins. The human sweet taste receptor is a heteromeric complex consisting of T1R2 and T1R3, both of which belong to the class C G-protein-coupled receptor family with a large extracellular domain [1], [2]. All of the aforementioned structurally diverse sweeteners are received by this receptor alone [3].

While the majority of sweet substances have low molecular weights, eight proteins are known to elicit sweetness: brazzein [4], lysozyme [5], [6], mabinlin [7], monellin [8], pentadin [9], thaumatin [10], miraculin [11], and neoculin [12], [13]. Among these proteins, neoculin, isolated from the edible fruit of *Curculigo latifolia* that grows in West Malaysia, induces an unusual taste sensation. Neoculin has a weak sweet taste of its own but also elicits intense sweetness shortly after tasting an acidic solution [14]. For example, the taste of sour lemon is sensed as a sweet, orange-like taste. This phenomenon, called taste modification, persists for 30–60 min each time after tasting a sour solution. Neoculin is currently the only known protein that both tastes sweet and has a taste-modifying activity. Miraculin, another taste-modifying protein, does not taste any sweet on its own and elicits intense sweetness after tasting acids [15]. Both taste-modifying proteins might be used as unique, non-glycemic taste improvers for sour foods.

**Figure 1:**
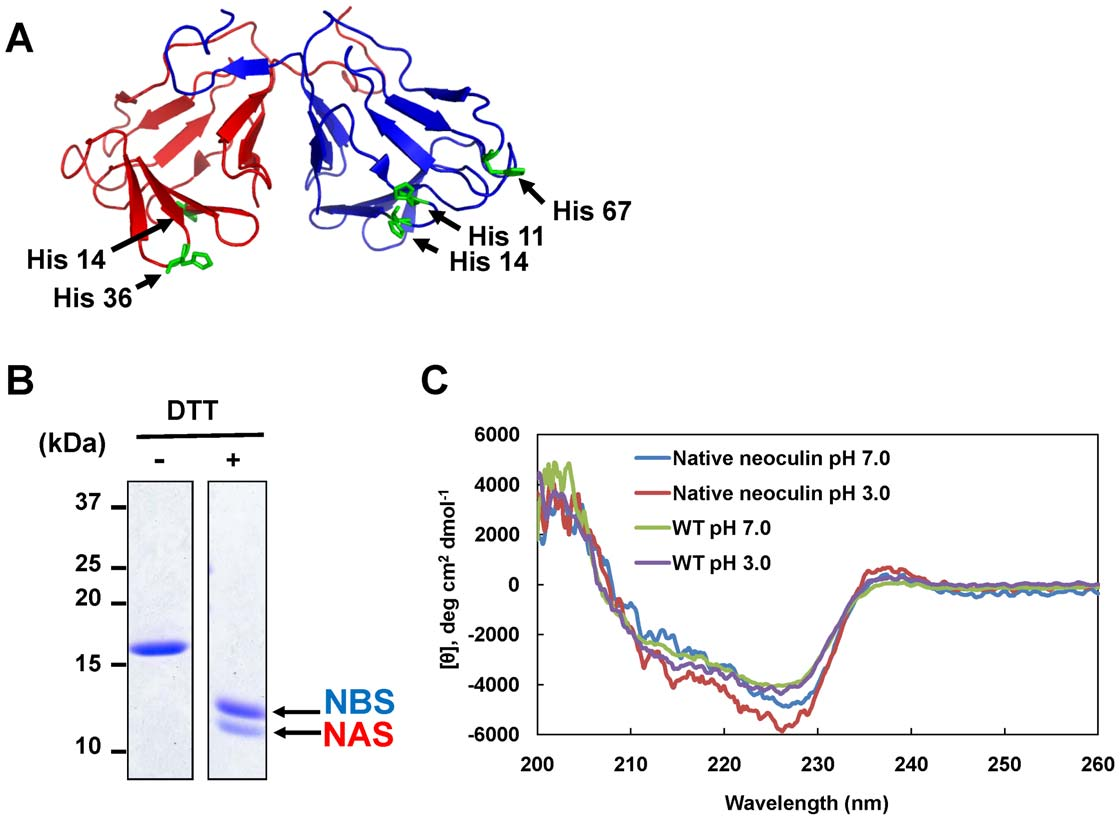
The crystal structure of neoculin (PDB ID: 2D04). (A) NAS and NBS are colored red and blue, respectively. His residues are shown with a green stick model. NAS has two His residues at positions 14 and 36. NBS has three His residues at positions 11, 14 and 67. (B) An SDS-PAGE analysis of bacterially produced wild-type neoculin. (C) Far-UV CD spectra of bacterailly produced and native neoculin samples. The far-UV CD spectra were recorded in 20 mm sodium phosphate buffer, pH 7 or 20 mM sodium citrate buffer, pH 3.0.

Structurally, neoculin is a clamshell-like heterodimer consisting of a neoculin acidic subunit (NAS) and a neoculin basic subunit (NBS), both of which are conjugated by two disulfide bonds (Fig. 1A [16]). We previously produced a “5HA” variant in which all five His residues of neoculin were converted to Ala using an *Aspergillus oryzae* expression system and found that this variant elicited strong levels of sweetness in a pH-independent manner, even at non-acidic pH [17]. These results indicate that the His residues of neoculin play an important role in its taste-modifying activity.

Which of the five His residues are critical for this taste-modifying activity? Are collective effects at work, or is only a single His residue needed? In this study, we performed a functional analysis of a series of His-to-Ala neoculin variants to answer these questions. We identified NBS His11 as a pH sensor that elicits the taste-modifying activity and produced a novel neoculin variant with miraculin-like activity by mutating this residue.

## Results

### Evaluation of the sweetness levels of neoculin variants produced by a bacterial expression system

To clarify the role of each His residue in the NAS-NBS heterodimer (Fig. 1A), a variety of neoculin variants were needed. Although we previously described an *Aspergillus oryzae*-based neoculin expression system [16], this system uses a time-consuming selection process to screen the heterodimer (active neoculin) -produced strain and was not suitable for the production of a large number of variants. Instead, a modified bacterial expression system, used by Suzuki *et al*., [13] was selected for this study. Briefly, each subunit was produced independently as an inclusion body. The individual purified subunits were mixed, refolded and dimerized, and the NAS-NBS heterodimer was separated from homodimers using cation exchange column chromatography. SDS-PAGE analysis of the purified heterodimer showed an 18-kDa single band under non-reducing conditions; the 11-kDa band of NAS and 13-kDa band of NBS appeared under reducing conditions (Fig. 1B). Similar band patterns and purities were observed for all of the neoculin variants used in this study (data not shown). A total of 10 mg of purified neoculin were obtained per 1 liter of culture. Far-UV circular dichroism (CD) spectroscopic analysis showed that bacterially produced and native neoculin samples have similar spectral patterns under both pH 3.0 and pH 7.0, suggesting that the recombinant neoculin was refolded just as the native protein (Fig. 1C).

We next examined whether bacterially produced wild-type (WT) neoculin and the 5HA variant (in which all five His residues in neoculin were replaced with Ala) had equivalent activity to the native protein and to the protein produced by *A. oryzae*, respectively [17].

**Figure 2:**
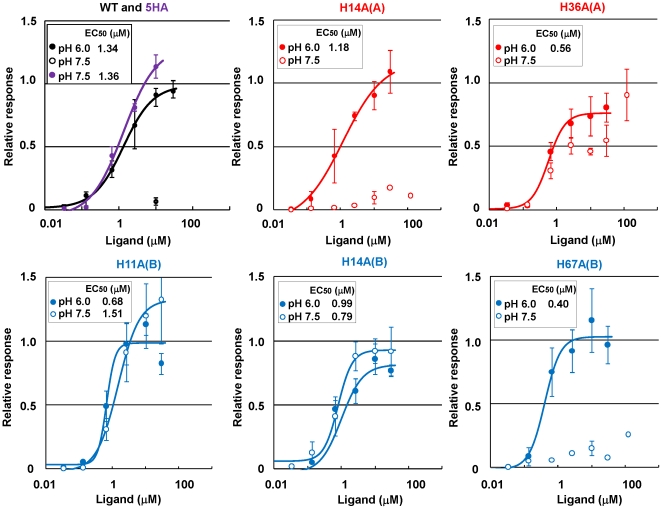
Dose-response relationships of His-to-Ala variants of neoculin in the cell-based assay. The responses of hT1R2-hT1R3 and G15Gi3 transfected cells were examined after the application of WT neoculin or its single point variants at weakly acidic pH (6.0; closed circle) and at neutral pH (7.5; open circle) and to that of the 5HA variant at neutral pH (7.5; purple circle). A neoculin variant with a His-to-Ala substitution at NBS position 11 is designated H11A(B). The number of responsive cells was normalized relative to the maximum response to aspartame (6.7 mM) at pH 7.4. Each point represents the mean ±SE from three or four independent experiments.

The cell-based assay was previously established to evaluate the acid-induced sweetness of neoculin quantitatively [17]. The human sweet taste receptor, T1R2 and T1R3 were transiently transfected into HEK293T cells together with G15Gi3, a chimeric Gα. Neoculin or its variant was applied to cells under weakly acidic (pH 6.0) and at neutral (pH 7.5) conditions (Fig. 2, closed and open circles, respectively).

The maximal relative response to bacterially produced WT neoculin was extremely weak at pH 7.5. It increased in a dose-dependent manner, reaching a relative response of approximately 1.0 at the half-maximal effective concentration (EC_50_) of 1.59 µM at pH 6.0. This value was similar to the EC_50_ of native neoculin [17]. The 5HA variant activated the receptor strongly even at pH 7.5, with an EC_50_ value of 1.36 µM (Fig. 2, purple), as was the case for the heterodimer produced by the *A. oryzae* expression system [17]. These results strongly indicate that the bacterially produced WT neoculin and 5HA variant have sweetness levels equivalent to the native protein and to that produced by *A. oryzae*, respectively.

### Dose-response relationships of single His-to-Ala variants in the cell-based assay

These results prompted us to produce five single His-to-Ala (HA) variants using the bacterial expression system (Fig. 1). To assess the dose-response relationships, each single HA variant was applied to cells at pH 6.0 and pH 7.5. As shown in Fig. 2, all five single HA variants activated the receptor strongly at pH 6.0, with EC_50_ values similar to that of WT neoculin. These results suggest that these variants and WT neoculin have similar sweetness levels at acidic pH. By contrast, the cellular responses to these variants at pH 7.5 could be categorized as weakly activating (H14A(A) and H67A(B)), modestly activating (H36A(A)), and strongly activating (H11A(B) and H14A(B)) (Fig. 2). These results suggest that not all five His residues are required for the pH-dependent activity of neoculin. NAS His14 and NBS His67 are not important for eliciting acid-induced sweetness; only NAS His36, NBS His11, and NBS His14 may be involved in this activity.

### Sensory properties of neoculin and its variants

Non-specific responses were often observed in the cell-based assay at pH values below 5.0 or above 7.6 (data not shown). To further clarify the pH-sensitive activities of neoculin variants, experimental conditions in a wider pH range than that of the cell-based assay should be carried out. We next evaluated the sweetness of neoculin and its variants in comparison with that of aspartame using a human sensory test. Panel members tasted neoculin or its variant solutions for 15 sec and scored their sweetness levels compared with those of standard aspartame solutions at three different concentrations (0.1 mM, 0.5 mM and 2.0 mM). Neoculin and its variants were dissolved in buffer solutions with one of three pH values: 4.0, 7.5, or 8.0.

**Figure 3:**
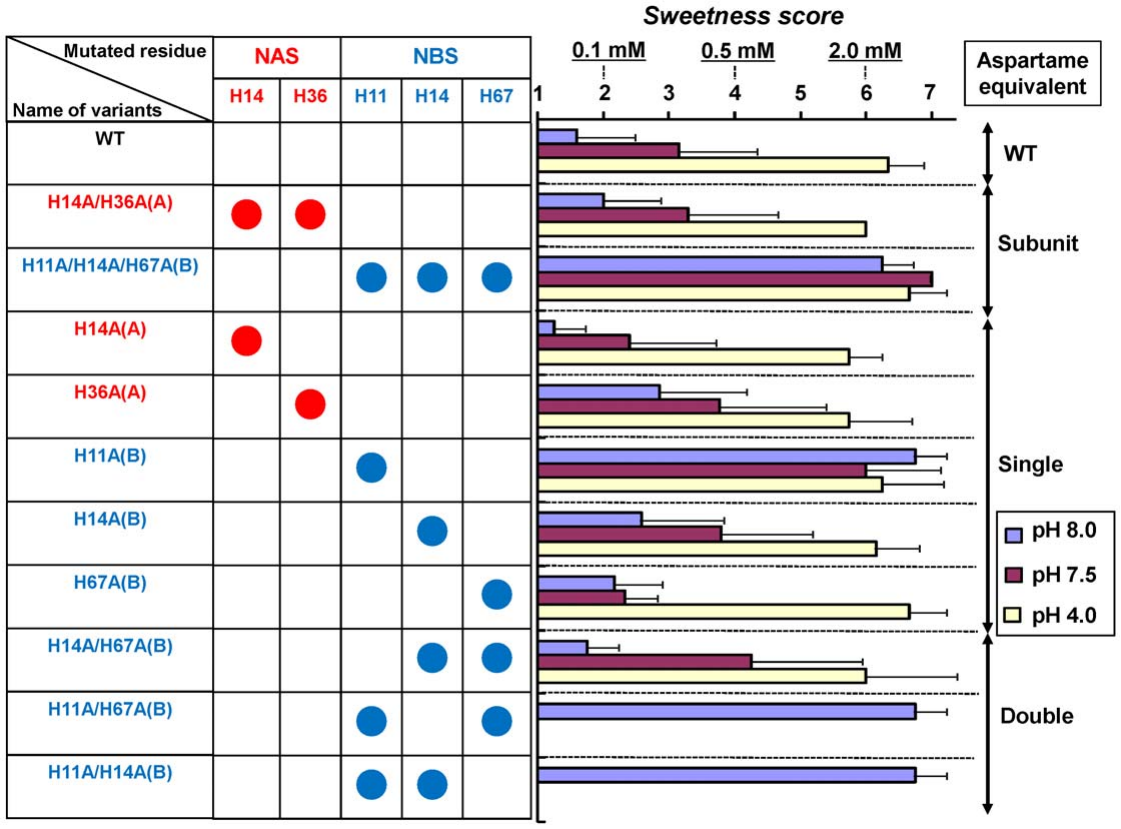
Human sensory test of His-to-Ala neoculin variants. Panelists tasted His-to-Ala neoculin variant solutions at pH 8.0 (blue), pH 7.5 (red) and pH 4.0 (yellow) for 15 sec and rated their sweetness. Each bar represents the mean ±SD (n = 4–10).

WT neoculin elicited almost no sweet taste (score: 1.6±0.84, mean ±SD) at pH 8.0, a weak sweet taste (score: 3.1±1.2) at pH 7.5 and a strong sweet taste (score: 6.3±0.58) at pH 4.0 (Fig. 3). These results demonstrate that WT neoculin elicits sweet taste in a pH-dependent manner. Next we determined which subunits contain a His residue critical for pH-dependent sweetness. Because NAS has two His residues at positions 14 and 36 and NBS has three His residues at positions 11, 14 and 67 (Fig. 1A), we produced H14A/H36A(A) and H11A/H14A/H67A(B) variants (Fig. 3, subunit). The former produced pH-dependent sweetness as WT neoculin; the latter had a strong sweet taste even at pH 8.0 (score: 6.3±0.5), as did the previously reported 5HA variant [17]. These results suggest that NBS contains the critical His residue(s).

To identify the critical His residue(s), the five single HA variants were evaluated. H11A(B) elicited a strong sweet taste under the three pH conditions tested. By contrast, the other four single HA variants elicited pH-dependent sweetness (Fig. 3, single). These results suggest that NBS His11 is specifically critical for the protein's pH-dependent activity. In addition, H36A(A) and H14A(B) tended to elicit modest sweetness at pH 8.0 (score for H36A(A): 2.9±1.4; score for H14A(B): 2.6±1.3) in contrast to the weak sweetness of WT neoculin at the same pH. These results indicate that NAS His36 and NBS His14 may be involved in the pH-dependent sweetness to a minor extent.

We finally evaluated whether NBS His11 was sufficient to elicit pH-dependent sweetness. A pH-independent sweet variant, H11A/H14A/H67A(B), was used as a template, and individual His residues were reinserted one by one. H14A/H67A(B) elicited pH-dependent sweetness, but the other two variants elicited intense sweetness even at pH 8.0 (Fig. 3, double). These results suggest that NBS His11 is sufficient to elicit the pH-dependent activity of neoculin.

### Modulation of pH-dependent activity by inserting point mutations of His11 into the NBS

**Figure 4:**
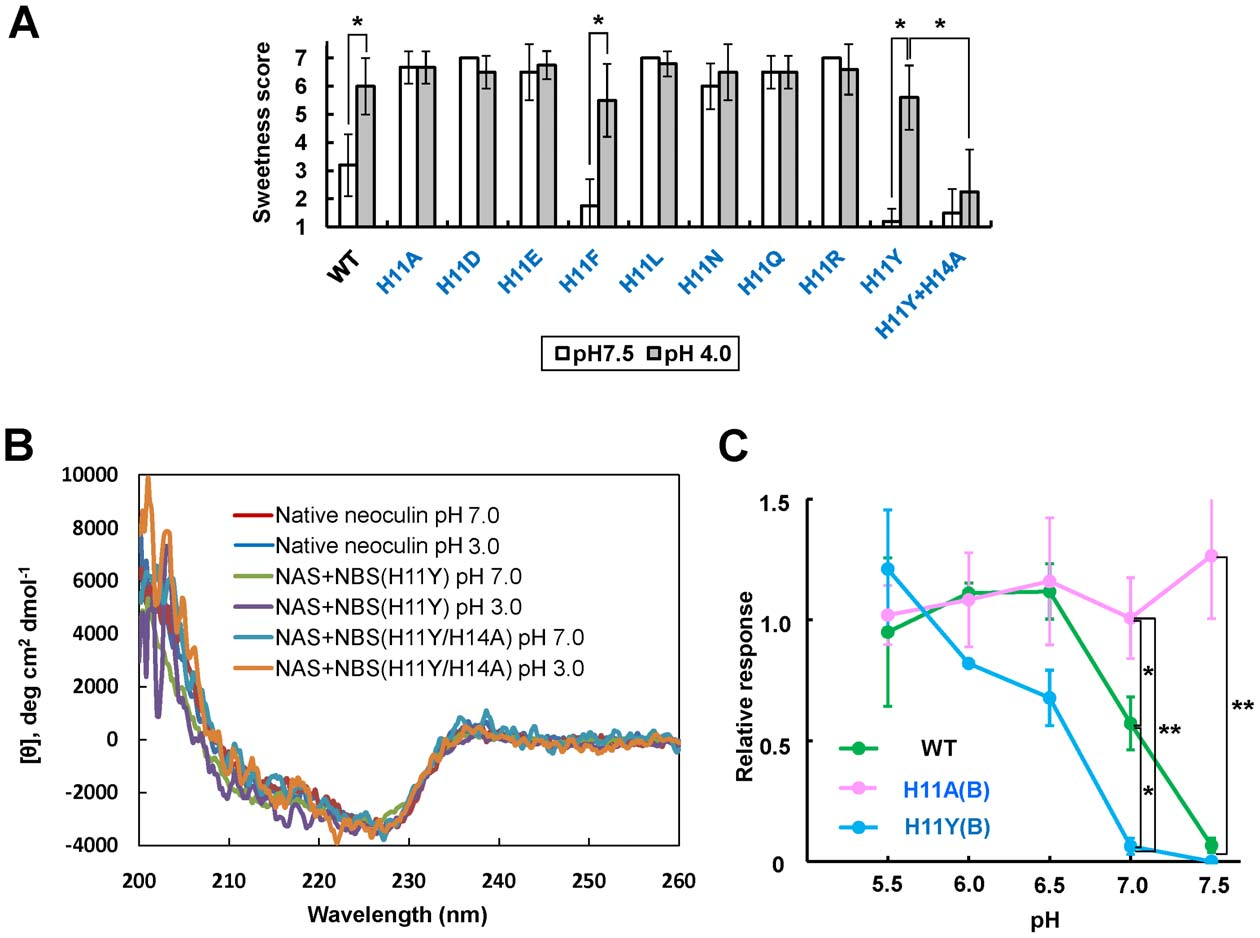
Modulation of the pH-dependent activity of neoculin. (A) Human sensory test of H11X(B) and H11Y/H14A(B) variants. Panelists tasted neoculin variant solutions at pH 7.5 (white) and pH 4.0 (grey) for 15 sec and rated their sweetness levels. Each bar represents the mean ±SD (n = 4–10). The sweetness score was analyzed with a Mann-Whitney *U*- test (*p<0.05). X indicates D, E, F, L, N, R,Q and Y. (B) Far-UV CD spectra of the native neoculin, H11Y(B) and H11Y/H14A(B). The far-UV CD spectra were recorded in 20 mm sodium phosphate buffer, pH 7 or 20 mM sodium citrate buffer, pH 3.0. (C) The pH-dependence of the cell response to neoculin variants. WT neoculin (green), H11A(B) (pink), or H11Y(B) (cyan) (10 µM each) was applied to cells expressing hT1R2-hT1R3 and G15Gi3 under pH 5.5–7.5. The number of responsive cells was normalized relative to the maximum response to aspartame (6.7 mM) at pH 7.4. *p<0.05 and **p<0.01, one-way ANOVA with Tukey's *post hoc* test. Each point represents the mean ±SE from three or four independent experiments.

To evaluate the role of NBS His11 in the pH-dependent activity of neoculin, we prepared NBS11-mutated variants other than His-to-Ala. Eight residues were selected on the basis of acidity/basicity, hydrophobicity and side chain size. In a human sensory test, the H11F(B) and H11Y(B) variants elicited pH-dependent activity with the loss of weak sweetness at neutral pH (score at pH 7.5: 1.8±0.96 for F and 1.2±0.45 for Y), similar to miraculin 15], but distinct from WT neoculin (score at pH 7.5: 3.2±1.1), while the other six variants showed strong pH-independent sweetness levels, as shown for H11A(B) (Fig. 4A). These results suggest that the NBS position 11 requires an aromatic amino acid such as Phe or Tyr to elicit the pH-dependent activity (as does the imidazole ring of His). The fact that His11(B) can be replaced by Tyr (protonatable but with shifted pKa) and Phe (not protonatable) suggests that not the protonation but the aromaticity itself at this site is critical. Interestingly, H11Y(B) elicited a pH-dependent activity, although this variant lost the pH-sensitive imidazole side chain (*pKa*  = 6.5). Because the side chain of Tyr (*pKa*  = 10) is not protonated in the pH range of 4.0–7.5, a pH-sensitive site other than His11 may be present in H11Y(B). We focused on NBS His14 because H14A(B) activated the receptor independently of pH in the cell-based assay (Fig. 2) and because this residue is close to NBS His11 in space (Fig. 1A). H11Y/H14A(B) was produced, and its sweetness level was scored. Although this variant is not different from native neoculin and H11Y(B) in overall structure, both of which had the taste-modifying activity (Fig. 4B), it largely lost its sweetness level at acidic pH (score at pH 4.0: 2.3±1.5) (Fig. 4A). This result suggests that NBS His14 might be involved in the pH-dependent activity of H11Y(B).

Finally, the pH-dependent activity of these variants was evaluated using a cell-based assay. Samples (10 µM) were applied to cells expressing hT1R2-hT1R3 in the pH range of 5.5–7.5. The relative response to H11A(B) was strongly pH-independent (Fig. 4C; pink); WT neoculin and H11Y(B) activated the receptor in a pH-dependent manner (Fig. 4C; green and cyan, respectively). On the other hand, there was a clear difference in the pH values eliciting the half maximal response between WT (pH 7.1) and H11Y(B) (pH 6.5) (Fig. 4C). This indicates that pH-sensitivity of acid-induced sweetness in neoculin can be controlled by the position 11 in NBS.

These results suggest that NBS His11 functions as a primary pH sensor for the taste-modifying activity and that engineering this residue leads to a novel taste-modifying protein with alternative pH sensitivity.

## Discussion

In this study, we found that not all five His residues of neoculin are necessary for its taste-modifying activity. NBS His11 is sufficient to induce pH-dependent sweetness. Moreover, we engineered a His-to-Tyr variant to create a novel taste-modifying protein with alternative pH sensitivity.

**Figure 5:**
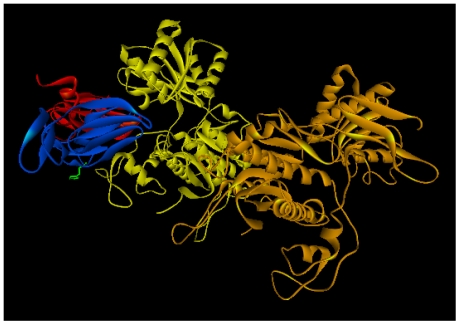
Docking model of neoculin and the extracellular domains of human sweet taste receptor. NAS, NBS, hT1R3, and hT1R2 are colored red, blue, yellow, and orange, respectively. NBS His11 is shown with a green stick model.

How does NBS His11 function in the taste-modifying activity of neoculin? One possibility is that His11 interacts with the sweet taste receptor directly to modulate its activation; the other is that His11 induces a conformational change in neoculin in a pH-dependent dynamic equilibrium between active and inactive forms, as described in our previous model [16]. To determine the precise mechanism, we reevaluated the previously constructed docking model of neoculin and the sweet taste receptor [16]. Taking into consideration of our previous experimental data [18], neoculin bound to the large extracellular domain of hT1R3 (Fig. 5). NBS His11 mapped on the model suggested that this residue is away from the interface between neoculin and the receptor (Fig. 5). It indicates that His11 does not interact directly with the receptor but instead changes the structure and activity of neoculin. Because there were clear differences in the pH sensitivity of WT, H11A(B) and H11Y(B) (Fig. 4C), it is strongly suggested that NBS position 11 is a critical point of control in the equilibrium between the protein's active and inactive forms.

While the position 11 in NBS required the aromatic amino acid, Tyr or Phe for eliciting pH-dependent sweetness, its replacement with one of acidic, basic, and hydrophobic amino acids induced pH-independent sweetness (Fig. 4A). We further evaluated the sensory properties of H11C(B). Although this variant was unique in that it contained a glutathione molecule conjugated via Cys11 during our refolding process, it had an intense sweetness, equivalent to H11A(B), in spite of its large side chain (data not shown). These results suggest that the unprotonated imidazole ring of His11 in NBS probably interacts with other residue(s) to keep the inactive form of neoculin under non-acidic pH conditions.

NBS His14 was important for eliciting the acid-induced sweetness of H11Y(B) since this variant retained its pH-dependent activity but H11Y/H14A(B) largely lost this activity (Fig. 4A). On the basis of the fact that His14 is close to His11 in the NBS structure (Fig. 1A), His11 and His14 may interact each other via their unprotonated imidazole rings to keep the inactive form of neoculin at neutral pH. A comparison of the structures of neoculin and NBS His11-mutated variants will help to define the precise roles and interactions between His11 and His14 in taste modification. Now we are performing X-ray crystal structural analysis of these proteins to clarify the molecular mechanism of pH sensing in neoculin.

WT neoculin activated hT1R2-hT1R3 in a pH-dependent manner at pH 7.1, eliciting a half-maximal response in the cell-based assay and it elicited a weak sweet taste at pH 7.5 in the human sensory test (Figs. 3, 4A, and 4C). On the other hand, H11Y(B) activated the receptor in a pH-dependent manner at pH 6.5, eliciting the half-maximal response, but it did not have any sweetness at pH 7.5 (Figs. 4A and C). These results suggest that the pH shift between the taste-modifying activity of WT and that of H11Y(B) is well correlated with their sweetness intensities.

In conclusion, we identified a primary pH sensor for the taste-modifying activity of neoculin. Insights into the molecular mechanism behind the pH-dependent activity of neoculin may lead to the development of novel taste-modifying proteins.

## Materials and Methods

### Native neoculin preparation

Purification of native neoculin from the fruits of *C. latifolia* (kindly provided by Dr. Toru Akita) was conducted as described previously [19].

### Mutagenesis

Neoculin has five His residues: two in the NAS and three in the NBS (Fig.1A). NAS and NBS cDNA variants were constructed using inverse PCR-based mutagenesis. Each cDNA was introduced into a pET21b expression plasmid at the *Nde*I and *Xho*I sites. Hereafter, the heterodimeric variant with a His-to-Ala substitution at NBS position 11 will be designated as H11A(B). Similar notation will be used for other variants.

### Expression and refolding of neoculin and its variant

NAS, NBS and their variant were produced using a bacterial expression system with slight modifications [13]. Each expression plasmid was transformed into *Escherichia coli* BL21 Codon Plus (DE3) RIL cells, and protein expression was induced with 3 mM isopropyl β-thiogalactoside. Bacterial pellets were resuspended in STE buffer (50 mM Tris-HCl, 10 mM EDTA, 150 mM NaCl, pH 8.0) and sonicated, and the precipitates were collected in inclusion bodies. The precipitates were washed three times with wash buffer (50 mM Tris-HCl, 1 mM EDTA, 2% Triton X-100, pH 8.0). After centrifugation, inclusion bodies were solubilized in 6 M guanidine hydrochloride, pH 2.0. The extinction coefficient of each subunit was calculated by adding the extinction coefficients of each tryptophan (5800 M-1) and tyrosine (1390 M-1). The protein concentration was determined by dividing the absorbance at 280 nm by the extinction coefficient. Aliquots (20 mg/ml) of both subunits were mixed together, diluted 20-fold under redox refolding conditions (50 mM Tris-HCl, 2 M guanidine-HCl, 0.285 mM oxidized glutathione, 2.85 mM reduced glutathione, pH 8.0), and incubated at 16°C for overnight to dimerize.

### Purification of neoculin and its variant

Each refolded protein was diluted with an equal volume of water and precipitated with 80% saturated ammonium sulfate at 4°C overnight. After centrifugation, the precipitated protein was dissolved in buffer A (20 mM acetate-Na, 100 mM NaCl, pH 4.5) and dialyzed against the same buffer. The dialysate was filtered through a 0.44-µm filter and chromatographed using a POROS HS cation exchange column (Applied Biosystems) with a linear gradient of 100–1200 mM NaCl in buffer A at a flow rate of 3.0 ml/min using a Waters 600 HPLC system (Waters, Milford, MA). Heterodimers were separated from reconstituted NAS-NAS and NBS-NBS homodimers that have no sweetness on their own and no taste-modifying activity [13]. Fractions containing the heterodimer were pooled, dialyzed against water, and lyophilized. The lyophilized protein was dissolved in a 150 mM NaCl solution, and purity of the protein was analyzed by SDS-PAGE.

### Far-UV CD spectra

CD spectra of neoculin and its variants were obtained in 20 mM sodium citrate buffer, pH 3.0 or 20 mm sodium phosphate buffer, pH 7.0 at 20°C using a J-720 spectropolarimeter (Jasco, Tokyo, Japan). Far-UV CD spectra were recorded at a protein concentration of 7.5 µm in a 1 mm cell at wavelengths in the range of 200- 260 nm. All spectra were corrected for absorbance of buffer. Two scans were averaged.

### Cell culture and calcium imaging

Cell culture and transfection were performed as described previously [17]. In brief, hT1R2 and hT1R3 were transfected into HEK293T cells (kindly provided by Dr. Hiroaki Matsunami, Duke University) together with G15Gi3, a chimeric Gα.

Calcium imaging was performed as described previously [17]. The number of responding cells was normalized relative to the maximum response to aspartame (6.7 mM) at pH 7.4. For the calculation of EC_50_ values, plots of amplitude *versus* concentration were prepared in Clampfit Version 9.2 (Molecular Devices). Nonlinear regression of the plots produced the function *f*(*x*) = *I*_min_+(*I*_max_−*I*_min_)/(1+(*x*/EC_50_)*^h^*), where *x* is the ligand concentration and *h* is the Hill coefficient, which was used to calculate the EC_50_ values for ligand-receptor interactions

We used 10 mM aspartame for the cell-based assay. This is because the degree of dispersion of cellular data were smaller when aspartame was used at 10 mM than at 2 mM, although hT1R2-hT1R3 transfected cells responded well to 2 mM aspartame which induced the intense sweetness in a human sensory test (See Fig.3).

### Human sensory test

A human sensory test was conducted with slight modifications [17]. The sweetness levels of neoculin and its variants were evaluated by panel members who had been trained to accurately describe sweetness intensity. In detail, they can discriminate the sweetness intensities of 300 µl each of aspartame solutions at three different concentrations—0.1 mM, 0.5 mM and 2.0 mM. Aspartame was dissolved in distilled water. For further accuracy, they fasted for more than 30 min before the sensory test. They first tasted 300 µl each of the standard solutions of aspartame at three different concentrations—0.1 mM, 0.5 mM and 2.0 mM—and noted their sweetness levels. The panel then tasted 150 µl of 20 µM neoculin or neoculin variant solution (50 mM citrate-NaOH, pH 4.0; 50 mM citrate-NaOH, pH 7.5; or 50 mM HEPES-NaOH, pH 8.0) for a period of 15 sec and were asked to rate its sweetness level.

The following 7-point scaling system was adopted: 7 for >2.0 mM, 6 for 2.0 mM, 5 for 0.5–2.0 mM, 4 for 0.5 mM, 3 for 0.1–0.5 mM, 2 for 0.1 mM, and 1 for <0.1 mM aspartame solution. All panelists gave written informed consent prior to the tests. The study was approved by the local ethics committee on human subjects at the University of Tokyo.

### Statistical data analysis

The data in Fig. 4A (n = 4–10) were tested for statistical significance by the Mann-Whitney *U*-test. The data in Fig. 4C (n = 3–4) were tested for one-way analysis of variance (ANOVA) with Tukey's *post hoc* test.

### Generation of docking model

The docking model was generated as described previously [16]. A structural model of the sweet-taste receptor T1R2-T1R3 was generated based on the crystal structure of the extracellular region of the metabotropic glutamate receptor (mGluR). The primary sequence of T1R2-T1R3 was obtained from the Swiss-Prot database (entry codes: TS1R2_HUMAN and TS1R3_HUMAN). The free form I structure of extracellular domain of mGluR (PDB entry: 1EWT) was used as a template.
